# Inhibition of tumor growth by cancer vaccine combined with metronomic chemotherapy and anti-PD-1 in a pre-clinical setting

**DOI:** 10.18632/oncotarget.23181

**Published:** 2017-12-08

**Authors:** Annacarmen Petrizzo, Angela Mauriello, Antonio Luciano, Domenica Rea, Antonio Barbieri, Claudio Arra, Piera Maiolino, Marialina Tornesello, Vincenzo Gigantino, Gerardo Botti, Gennaro Ciliberto, Franco M. Buonaguro, Maria Tagliamonte, Luigi Buonaguro

**Affiliations:** ^1^ Laboratory of Molecular Biology and Viral Oncology, Istituto Nazionale per lo Studio e la Cura dei Tumori, Fondazione Pascale, IRCCS, Naples, Italy; ^2^ Animal Facility, Istituto Nazionale per lo Studio e la Cura dei Tumori, Fondazione Pascale, IRCCS, Naples, Italy; ^3^ Pharmacy Unit, Istituto Nazionale per lo Studio e la Cura dei Tumori, Fondazione Pascale, IRCCS, Naples, Italy; ^4^ Unit of Pathology, Istituto Nazionale per lo Studio e la Cura dei Tumori, Fondazione Pascale, IRCCS, Naples, Italy; ^5^ Scientific Directorate, Regina Elena National Cancer Institute, Rome, Italy

**Keywords:** cancer vaccine, mutated epitopes, metronomic chemotherapy, anti PD-1, combinatorial strategy

## Abstract

Tumor microenvironment (TME) is characterized by multiple immune suppressive mechanisms able to suppress anti-tumor effector cell immunity. Combinatorial strategies, including vaccine and immunomodulatory drugs, need to be developed for improved immunotherapy efficacy.

A novel combinatorial approach was assessed in C57BL/6 mice injected with mouse melanoma B16F10 cells. A multi-peptide vaccine (PEPT) was combined with a low dose metronomic chemotherapy (MCT) and an anti-PD-1 checkpoint inhibitor (CI). Statistical analysis were performed with the unpaired two-sided Student’s *t*-test and ANOVA.

Animals treated with the multi-peptide vaccine combined with MCT or CI showed remarkable delay in tumor growth and prolonged survival as compared to control groups. The multi-pronged combination including PEPT+MCT+CI was able to prolong survival in all mice and inhibit tumor growth in 66.6% of mice. All animals which did not show tumor growth were re-challenged with the same melanoma cells and one of them showed complete tumor growth inhibition. The anti-tumor effect was associated with strong T cell immune response to vaccine mutated peptides and significant reduction of regulatory T cells.

The combination of a vaccine with MCT and CI was highly efficient in potentiating the vaccine’s anti-tumor effects. The approach is highly promising to be moved into clinical trial.

## INTRODUCTION

Anti-tumor immunity is severely hindered by multiple immune suppressive mechanisms characterizing the tumor microenvironment (TME). As a result, tumor cells are able to escape the control operated by the immune system.

The main immune suppressive cell types in TME, among others, are represented by CD4^+^CD25^+^FoxP3^+^ regulatory T cells (Tregs). Tregs are able to lower antitumor immunity by suppressing NK and effector T cell responses [1–4]. Consequently, their percentage is inversely correlated with tumor progression and poor prognosis [5–8]. Additionally, the antitumor immune response is profoundly inhibited by immune checkpoint molecules (i.e. programmed death ligand-1, PD-L1) expressed on cancer cells. The binding of PD-L1 to programmed death 1 (PD-1) receptor on the surface of activated T and B cells, induces inhibitory pathways and generates a net immunosuppressive effect. This allows tumor evading cell killing [9]. Different tumors express PD-L1 molecules and, in recent years, treatment with blocking monoclonal antibodies has been shown to induce tumor regression in significant percentage of cancer patients (reviewed in [10, 11]).

Overall, the intra-tumor infiltration of immune suppressive cell types, combined with the inhibitory signals provided by the immune checkpoint molecules expressed by cancer cells, makes the TME highly unfavorable to immunotherapies. Indeed, it is one of the major reason for the unsatisfactory results observed in cancer vaccine clinical trials [12].

Consequently, in order to dramatically improve the immune response by cancer vaccines, TME needs to be corrected by therapeutic treatments [13].

Chemotherapy agents, administered at standard or low-dose metronomic regimens have been shown to selectively kill immunosuppressive cell populations. Moreover, they induce an immunogenic cell death in cancer cells with the release of danger signals which polarize dendritic cells (DCs) towards a pro-inflammatory phenotype driving an anti-tumor T helper 1 (Th1) response (reviewed in [14]). This would eventually generate a favorable tumor immune environment and potentiate effects of anticancer vaccines (reviewed in [15, 16]). Similarly, combination between checkpoint inhibitors and cancer vaccines have been reported in pre-clinical settings and in two clinical trials, showing significant enhancement in vaccine induced immune response and anti-tumor effects [17–24]. Alternatively, combination of anti-CTLA-4 (Cytotoxic T-Lymphocyte Antigen 4) and metronomic chemotherapy has been recently shown to be effective in a breast cancer pre-clinical model [25]. Furthermore, combination of tumor-antigen-targeting antibody, recombinant interleukin-2, anti-PD-1 and a powerful T cell vaccine has been recently shown to be extremely promising against B16F10-derived tumor [26].

In this framework, we have previously reported a novel combinatorial strategy, based on vaccine and a metronomic chemotherapy including taxanes and alkylating agents. The combinatorial strategy induced an enhanced vaccine-specific T cell response in a tumor-free setting, when compared to vaccine alone, which correlated to a reduced Treg frequency [24]. The same MCT strategy was subsequently shown to significantly delay tumor growth, prolong animal survival but was not sufficient to provide full protection. Such anti-tumor biological effects directly correlated with induction of immunological cell death, enhanced T cell response and reduction of the immune suppressive Tregs cell population [27].

In the present study, the overall objective was to evaluate the enhanced anti-tumor immunological effects of a vaccine (PEPT) when combined with metronomic chemotherapy (MCT) and a checkpoint inhibitor (CI). The experimental tumor model was based on sub-cutaneous orthotopic implantation of B16F10 melanoma cells in C57BL/6 mice.

All the different combinations were shown to be safe, well tolerated and highly effective inducing delayed tumor growth and improved survival, compared to the vaccine alone. In particular, a progressive improved survival over control groups was observed. Moreover, tumor growth was completed inhibited in 50% of animals treated with PEPT+MCT and, remarkably, in 66% of animals treated with the triple PEPT+MCT+CI combination. One of the animals from the latter group was fully protected and remained tumor negative also after a subsequent tumor re-challenge. Such results show a great improvement of anticancer vaccine efficacy.

## RESULTS

### Effect of combinatorial strategies on tumor growth and mice survival

C57BL/6 mice were divided in 7 groups (6 animals per group) and treated as described in Supplementary Figure 1. The general status of animals in the experimental groups was monitored during the whole protocol. No toxicity was observed, all animals showing good general status without any significant weight loss (data not shown).

**Figure 1:**
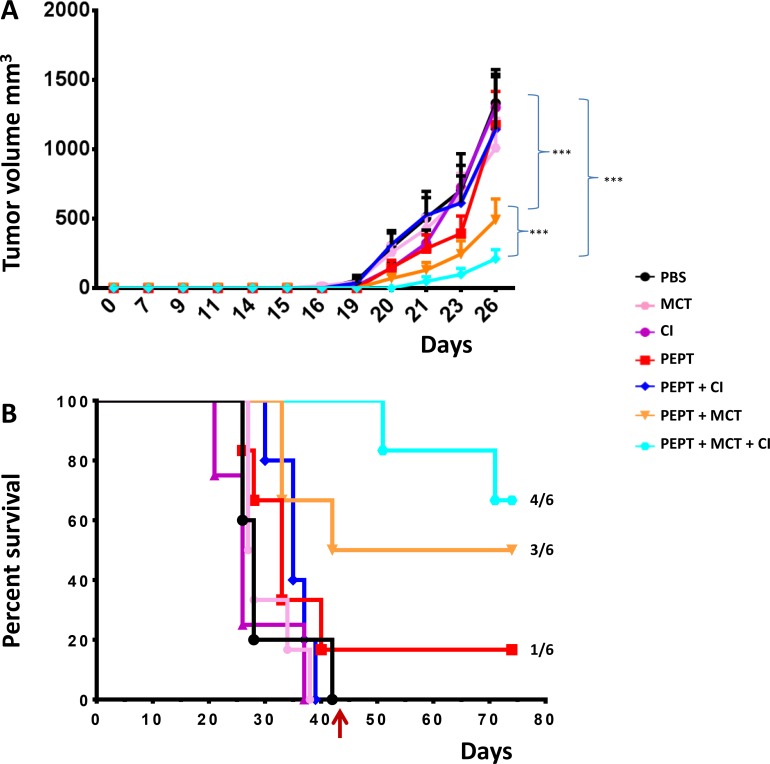
C57BL/6 mice were injected with 1 × 10^5^ cells in the right flank (**A**) Tumor growth was evaluated every two days with a caliper and tumor volume was calculated as indicated in Materials and Methods. Animals were sacrificed when tumor volume was greater than 1500 mm^3^. (**B**) Kaplan-Meyer curve showing the percentage of survival of animals in the experimental groups.

When the tumor reached the cutoff of 1500 mm^3^ in the first animal of the control group (day 26), tumor volume in all the different groups was compared. The results showed that all the combinatorial strategies induced a significant delay in tumor growth when compared to control and peptide vaccination only (*p* < 0.01) (Figure 1A).

Considering the whole observation period, the peptide vaccination alone was unable to control tumor growth in 5/6 animals with a pattern comparable to animals in the control group. However, 1 out of 6 animals showed complete control of tumor growth and survived until the end of the protocol (day 77) (Figure 1B). The combination with weekly MCT (PEPT+MCT) improved the antitumor effect of the vaccine with a 27% increment in the median survival. 50% of the animals showed complete control of tumor growth and survived until the end of the protocol (Figure 1B). On the contrary, combination with CI (PEPT+CI) induced a delay in tumor growth but did not improved survival compared to the vaccine alone (Figure 1B).

The best result was observed in the group treated with the vaccine combined with MCT and CI (PEPT+MCT+CI), in which the survival remained always above the 50%. Indeed, tumor growth was controlled in all animals until the treatment was discontinued. After that, two animals showed tumor growth, while 4/6 animals (66.6%) showed complete control of tumor growth and survived until the end of the protocol (day 77) (Figure 1B).

### Combinatorial strategies induce a reduction of Treg population

In order to clarify the immunological mechanisms underlying the delay in tumor growth observed in the animal groups treated with the different combinations, Tregs were evaluated in blood, resected spleens and LN.

**Figure 2:**
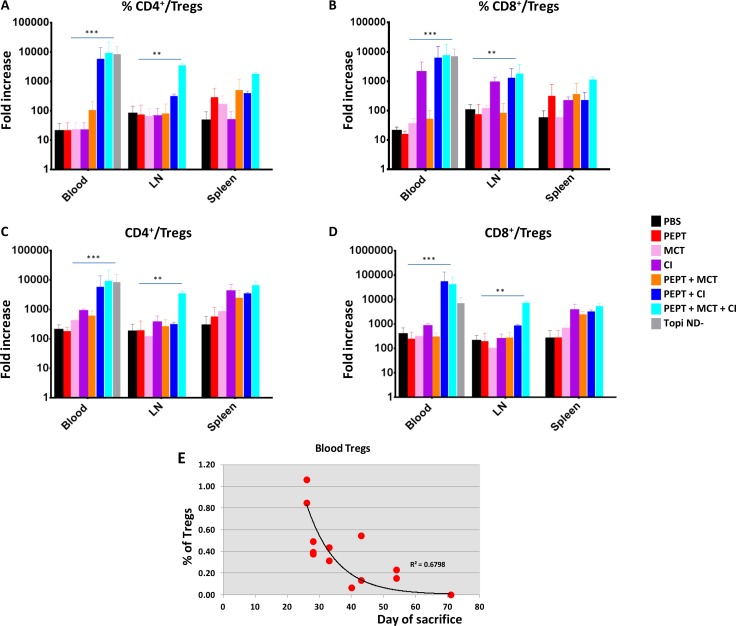
Evaluation of T_CD4_^+^/T_reg_ and T_CD8_^+^/T_reg_ ratio in blood, LN and spleens in the experimental groups Ratio between percentages (**A** and **B**) ; ratio between absolute numbers (**C** and **D**). Correlation analysis between Tregs in blood and day of sacrifice for each animal (**E**).

All combinatorial strategies, when compared to the vaccine alone, did not have any impact on CD4^+^ and CD8^+^ Tcells (Supplementary Figures 2 and 3). On the contrary, they induced a reduction in the CD4^+^CD25^+^FoxP3^+^ Treg population in the three compartments although the effect was much more evident in the blood (Supplementary Figures 2 and 3). Moreover, the most dramatic reduction was observed in animals treated with CI, alone or in combination with MCT. Interestingly, the tumor-negative mice (TN mice) showed the lowest percentage of Tregs (Supplementary Figures 2 and 3). Such result was further supported by the T_CD4_^+^/T_reg_ and T_CD8_^+^/T_reg_ ratio which showed a significant increase in all experimental groups treated with combinatorial strategies, compared to the vaccine alone. As for the Treg evaluation, such increase was more pronounced in animals treated with CI, alone or in combination with MCT, in the blood and LN compartments as well as in TN mice (Figure 2A–2D). A significant inverse correlation was observed between animal survival and percentage of Tregs in the blood compartment (Figure 2E).

### Evaluation of IFN-γ producing T cells

**Figure 3:**
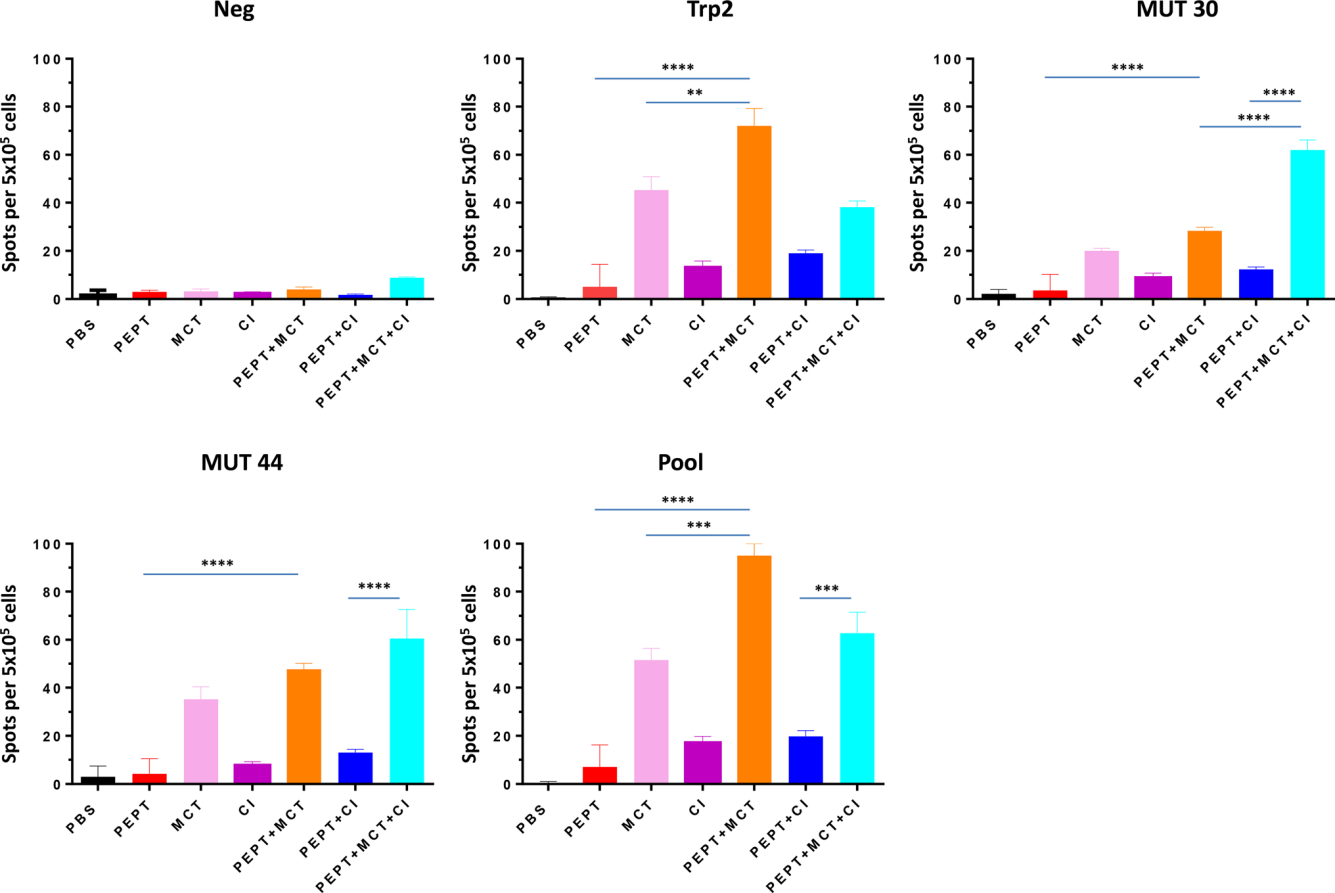
IFN-γ ELISPOT obtained in splenocytes reported as mean from animals of each experimental group Results are expressed as number of spots per 5 × 10^5^ splenocytes. NEG stands for PBS and POOL stands for pooled vaccine peptides.

T cell reactivity against vaccine peptides was assessed in splenocytes by IFN-γ Elispot assay. Vaccine alone induced only a modest T cell response specific for the vaccine peptides. Splenocytes from mice treated with PEPT+MCT combination showed a considerable increase of IFN-γ production compared to either the vaccine or the MCT alone. In particular, it was significantly stronger to wt Trp2 peptide than to mutant peptides (*p* < 0.05) (Figure 3). On the contrary, splenocytes from animals treated with PEPT+CI combination showed only a modest increase in IFN-γ production without statistical significance. Interestingly, the PEPT+MCT+CI combination induced the strongest T cell response targeting the two mutant peptides (Figure 3). A significant correlation was observed between animal survival and number of IFN-γ spots, which reached the highest values for the mutant peptides (Supplementary Figure 4).

### Multi-parametric evaluation of anti-tumor immune responses

**Figure 4:**
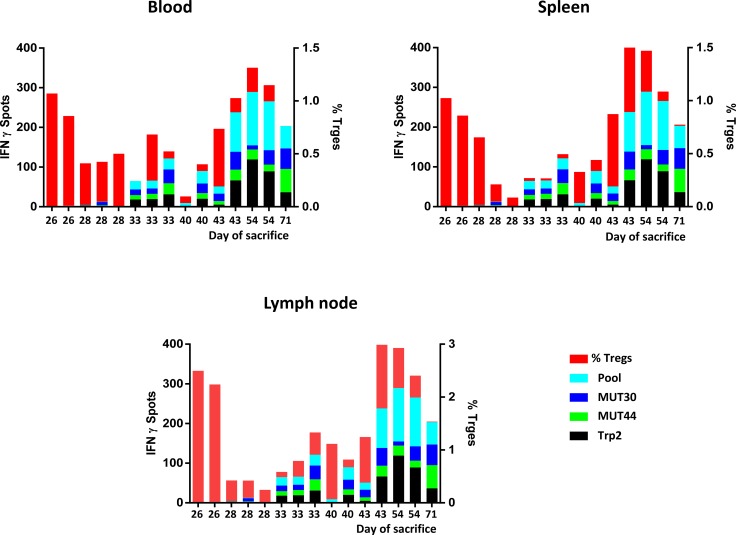
Cumulative multiparametric representation per individual mice including number of IFN-γ spots, percentage of Tregs and day of sacrifice in the three compartments

The pattern of active (e.g. antigen-specific IFN-γ spots) and regulatory (e.g. Tregs) anti-immune responses in each animal, irrespective of the experimental group, was correlated with the day of sacrifice. Overall, the percentage of Tregs was much higher in animals unable to control tumor growth (e.g. earlier sacrifice). Inversely, the number of antigen-specific IFN-γ spots was much higher in animals controlling tumor growth (e.g. later sacrifice). Notably, the animal from the PEPT+MCT+CI experimental group able to control tumor growth for the longest period (77 days) showed almost undetectable Tregs and the highest number of IFN-γ spots specific for the two mutated epitopes (Figure 4).

### Analysis of tumor-infiltrating effector cells seen in all tumor models

**Figure 5:**
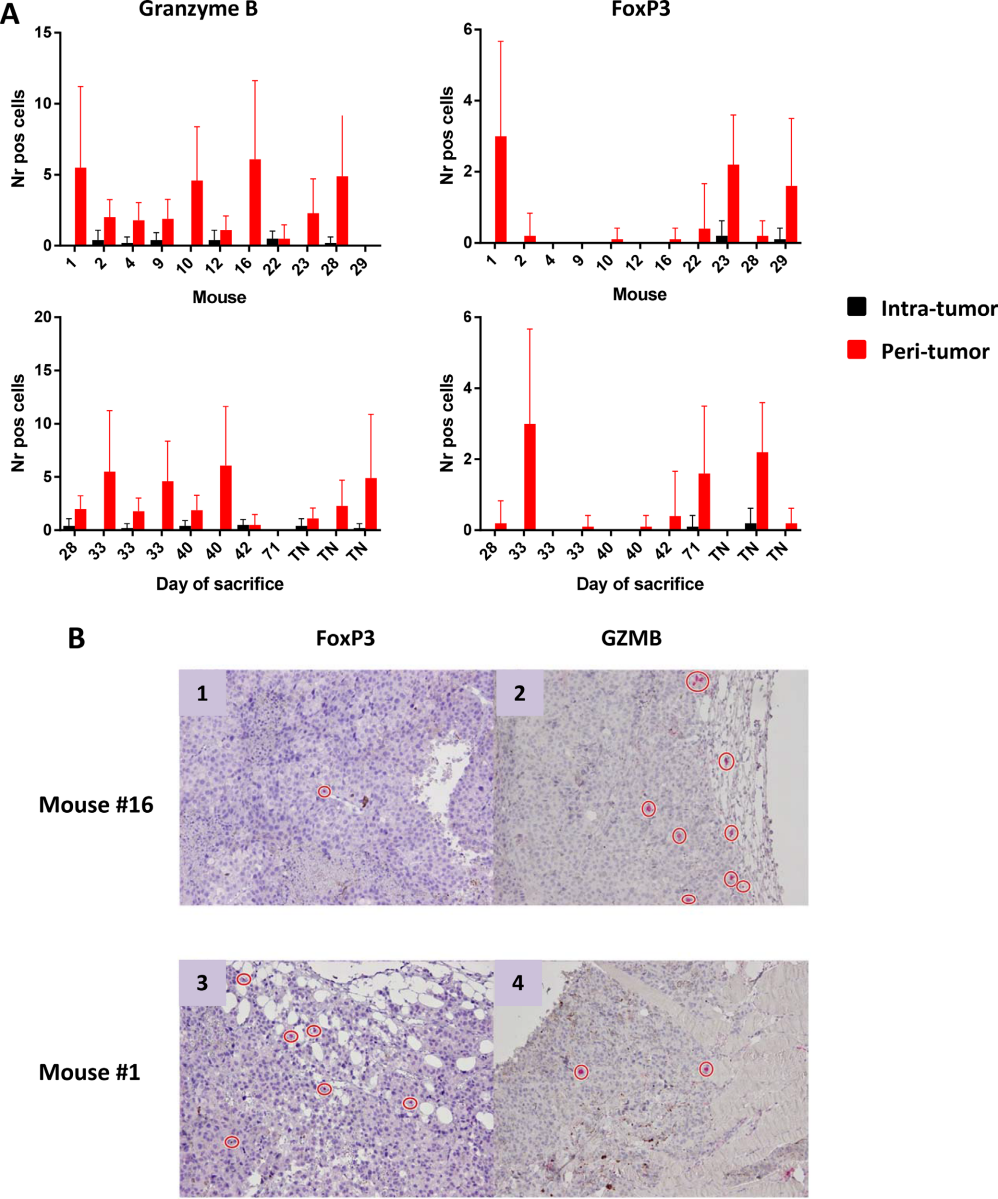
Number of Granzyme B^+^ and FoxP3^+^ cells infiltrating tumors (**A**) Cells are represented as mean with standard deviation (SD) in individual mice (upper panels) and according to day of sacrifice (lower panels). Ten different fields were counted. TN = tumor negative mice. FoxP3^+^ and Granzyme B^+^ cells in peri-tumoral area by IHC (**B**) Representative images of IHC staining of FoxP3^+^ and Granzyme B^+^ cells in the peri-tumoral areas in two mice characterized by low FoxP3^+^ and high Granzyme B^+^ (panels 1 and 2) or high FoxP3^+^ and low Granzyme B^+^ (panels 3 and 4).

The number of effector and regulatory cells in tumor sections from tumor-bearing mice was assessed using immunohistochemistry. IHC staining showed rare or absent CD3^+^ T cells. The absolute number of Granzyme B^+^ (GZMB) as well as FoxP3^+^ cells were very low and mostly confined at the peri-tumoral areas (Figure 5). No correlation was observed between numbers of infiltrating cells and tumor progression, expressed as day of sacrifice. Cells indicated for TN mice have been counted in tumors developed after the re-challenge.

### Tumor re-challenge in tumor-negative animals

**Figure 6:**
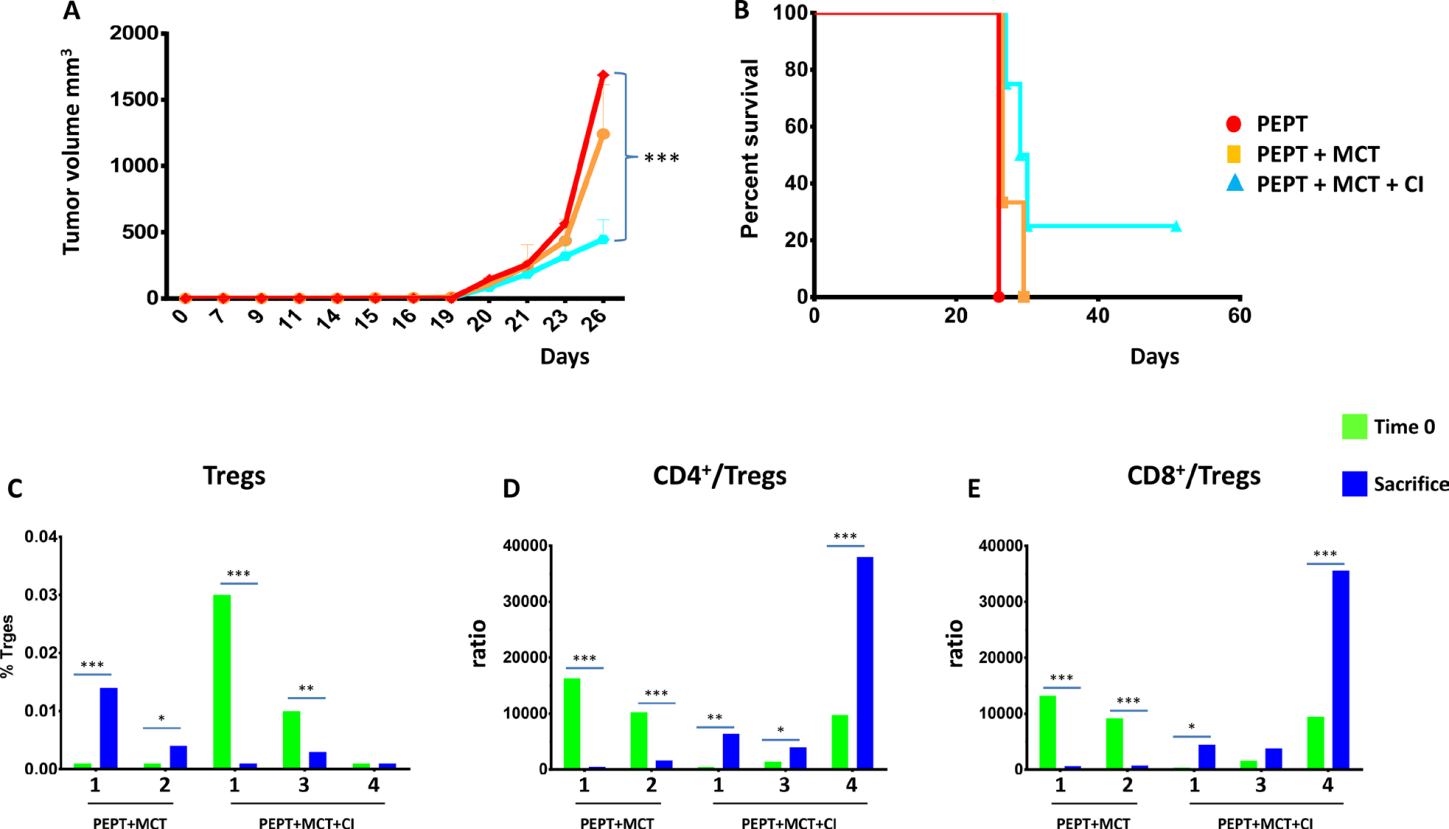
TN mice were re-challenged with 1 × 10^5^ cells in the left flank (**A**) As for the first immunization protocol, animals were monitored every 2 days for tumor growth and sacrifice occurred when tumor volume reached 1500 mm^3^. When the tumor reached the cutoff in the first animal (day 26), tumor volume in all the different groups was compared. (**B**) Kaplan-Meyer curve showing the percentage of survival of animals in the experimental groups. Days are from the re-challenge. Evaluation of CD4^+^ CD25^+^FoxP3 T regulatory cells (**C**) and T_CD4_^+^/T_reg_ and T_CD8_^+^/T_reg_ ratio (**D** and **E**) in the blood of each re-challenged animal at day 0 (green) and at sacrifice (blue).

TN mice from the experimental groups PEPT, PEPT+MCT and PEPT+MCT+CI were re-challenged with B16F10 melanoma cells without any further treatment. When the tumor reached the cutoff in the first animal (day 26), tumor volume in all the different groups was compared. The results showed a significant delay in tumor growth in animals coming from the PEPT+MCT group and even more significantly in those coming from the PEPT+MCT+CI group, when compared to animals originally treated with PEPT (*p* < 0.01) (Figure 6A).

Considering the whole observation period, animals from the PEPT and the PEPT+MCT groups were unable to control tumor growth, although the MCT slightly improved the antitumor effect of the vaccine (Figure 6B). On the contrary, animals from the group originally treated with PEPT+MCT+CI showed a slightly delayed tumor growth in 3/4 animals. The last animal of the group, even in the absence of any further immunization, showed complete control of tumor growth and was tumor-negative until the end of the protocol (Figure 6B).

### Protective memory immunity against tumor challenge

In order to clarify the immunological mechanisms underlying the pattern of tumor growth and survival in the animals re-challenged with the tumor, CD4^+^ and CD8^+^ T cells as well as Tregs were evaluated in blood. The level of Tregs at the day of the challenge (Day 0) was very low in all TN mice, ranging from 0.001 to 0.03%, and the T_CD4_^+^/T_reg_ and T_CD8_^+^/T_reg_ ratio was high (8 × 10^3^ on average), with significant variation among individual animals (Figure 6C, 6D, 6E). At the time of sacrifice, the percentage of Tregs as well as the value of T_CD4_^+^/T_reg_ and T_CD8_^+^/T_reg_ ratio was notably different in each animal (Figure 6C, 6D, 6E), strongly correlating with the day of sacrifice (R^2^ > 0.94) (data not shown). Indeed, animals dying earlier (#1 and 2_PEPT+MCT_) showed an increase in the percentage of Tregs as well as a dramatic reduction of the T_CD4_^+^/T_reg_ and T_CD8_^+^/T_reg_ ratio. Animals dying later (#1 and 3_PEPT+MCT+CI_) showed a significant reduction in the percentage of Tregs, which however was quite high at time 0. In parallel, the T_CD4_^+^/T_reg_ and T_CD8_^+^/T_reg_ ratio was found increased at the day of sacrifice. Interestingly, the animal #4_PEPT+MCT+CI_, which remained tumor negative for the entire re-challenge experiment (up to day 50), showed no increase in the percentage of Tregs and a relevant increase in the T_CD4_^+^/T_reg_ and T_CD8_^+^/T_reg_ ratio (> 3.5 × 10^4^) (Figure 6C, 6D, 6E).

### Evaluation of IFN-γ producing T cells in tumor challenge experiment

**Figure 7:**
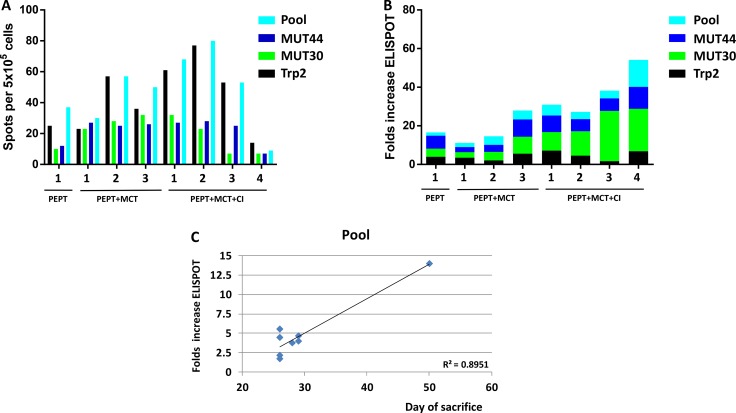
(**A**) Evaluation of interferon-γ (IFN-γ) secreting T cells after *in vitro* re-stimulation with individual or pooled vaccine peptides in individual TN mice at the day of re-challenge. (**B**) Fold increase of number of IFN-γ spots between T0 (day of re-challenge) and day of sacrifice in individual TN mice. (**C**) Correlation analysis between day of sacrifice of each animal and number of spots.

The specific response to vaccine peptides was evaluated in mice before the re-challenge (time 0) and at time of sacrifice on circulating PBMCs. As expected, at time zero all animals showed significant number of IFN-γ spots against the individual and pooled vaccine peptides, although they were highly variable among the different animals. On average, the strongest response was observed against the Trp2 peptide and the pool of peptides (Figure 7A). At the day of sacrifice, the increase in number of spots against the individual and pooled vaccine peptides was dramatically different in the individual animals. In particular, such increase was much more evident when T cells were re-stimulated with the two mutant peptides, especially in animals deriving from the group originally treated with PEPT+MCT+CI (Figure 7B). A significant correlation was observed between animal survival and IFN-γ number of spots, which reached the highest values for the pooled peptides (R^2^ = 0.89) (Figure 7C). Comparable results on IFN-γ number of spots were observed on splenocytes obtained from animals at time of sacrifice (data not shown).

### Overall analysis of the fully protected mouse #4_PEPT+MCT+CI_

**Figure 8:**
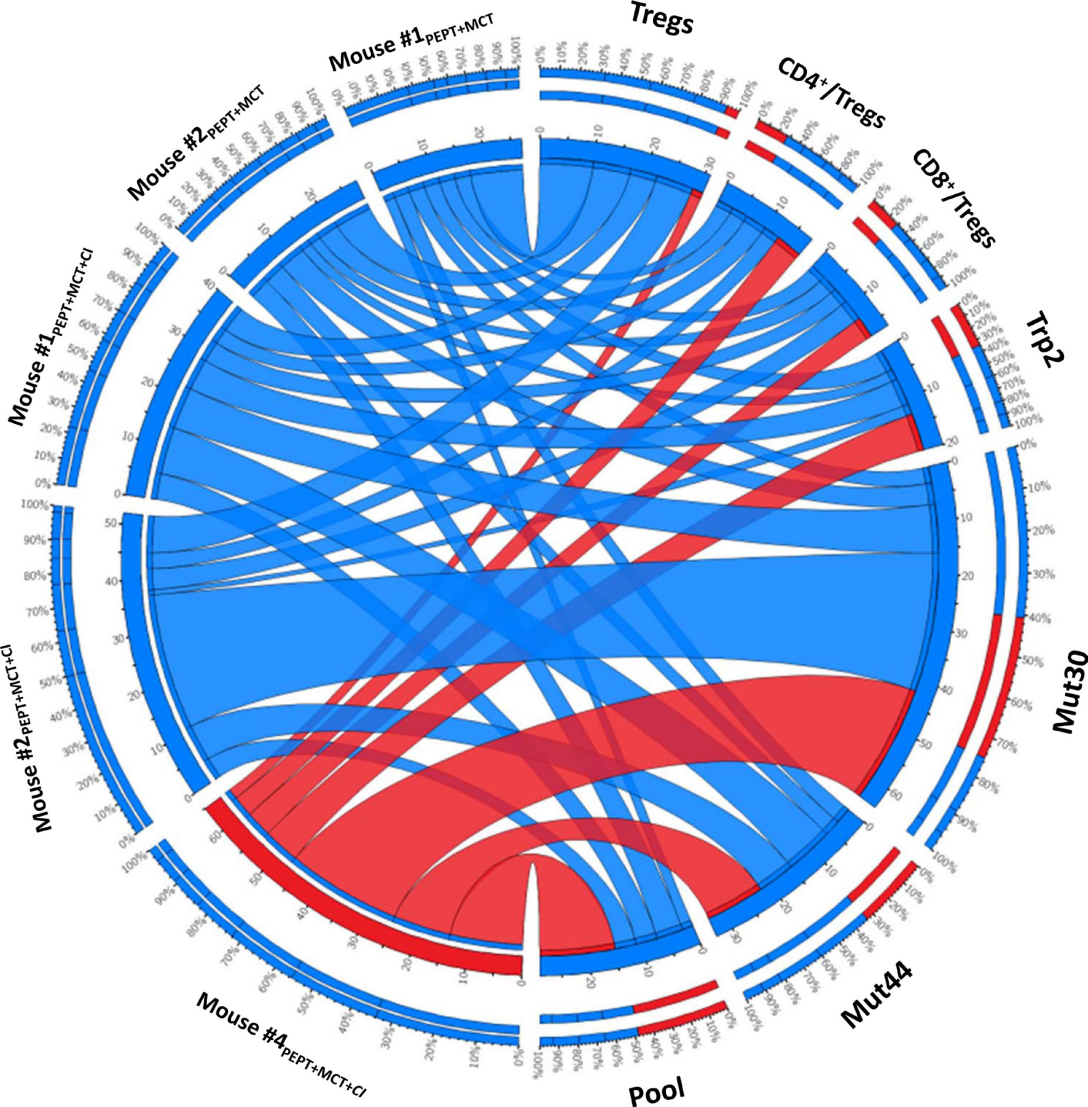
Cumulative multiparametric circos representation per individual re-challenged mice including number of IFN-γ spots specific for individual and pool of peptides, percentage of Tregs and T_CD4_^+^/T_reg_ and T_CD8_^+^/T_reg_ ratio The width of the links connecting each sample with each parameter indicates the actual value of that specific parameter. The inner ring indicates the absolute value; the outer ring is the percentage for that parameter. Data related to the full protected mouse #4_PEPT+MCT+CI_ are highlighted in red.

In order to better visualize the characteristics of the full protected mouse #4_PEPT+MCT+CI_, data from the different immunological parameters were combined in a Circos representation. Indeed, such a representation allows to have in the same graph all the parameters for each sample. Each animal undergoing the re-challenge experiment has been plotted against all the indicated immunological parameters (Figure 8). The cumulative multiparametric representation clearly indicated that the mouse #4_PEPT+MCT+CI_ showed the lowest percentage of Tregs, the highest T_CD4_^+^/T_reg_ and T_CD8_^+^/T_reg_ ratio as well as the highest number of IFN-γ spots specific for the Trp2, MUT44 and pool peptides and the second highest number of IFN-γ spots specific for the MUT30 peptide (Figure 8).

## DISCUSSION

In the current study, the combination of a cancer vaccine with different immune modulating treatments was investigated in a C57BL/6 animal model injected with the highly aggressive B16F10 melanoma cell line. Among different potential targets specific for B16 melanoma cell line [28], the cancer vaccine was a peptide mix including the melanoma-specific Tyrosinase-related protein 2 (Trp2_180–188_) and two mutated peptides (e.g. MUT30 and MUT44) [29]. It was combined either with a daily or weekly administered multi-drug metronomic chemotherapy (MCT) previously described by us [27], or with a PD1 inhibitor (CI), alone or in association with the weekly MCT. The rationale was to verify whether the anti-tumor immunity elicited by the cancer vaccine was potentiated by combination with immune modulatory drugs contrasting different components of the intratumor immune suppressive environment.

The results showed that all the combinatorial strategies induced a significant delayed tumor growth when compared to control and peptide vaccination only, with an increased median survival > 27% (Figure 1A). The vaccination alone was successful in inducing tumor growth inhibition in one animal. The combination with anti-PD-1 (PEPT+CI) did not improve neither median survival nor inhibited tumor growth. Such result was somehow expected given the poor immunogenicity of B16 derived tumors [30] and the reported unresponsiveness to checkpoint inhibitors [31, 32].

The combination of the vaccine with the weekly administered MCT significantly potentiated such effect, resulting in a tumor growth inhibition in 50% (3/6) of the animals (Figure 1B). Notably, tumor growth in mice treated with the combination including the vaccine with MCT and CI was remarkably delayed in all six mice (100%) and inhibited in 66.6% (4/6) of the animals (Figure 1B). These findings clearly showed that a multi-pronged inhibition of the immune suppressive intra-tumoral micro-environment dramatically potentiate the anti-tumor efficacy of the vaccine.

All combinatorial strategies induced a trend of increase for both CD4^+^ and CD8^+^ T cells in the PBMCs, when compared to the vaccine alone, which significantly correlated with the day of sacrifice of the corresponding animal. Such a correlation was not observed with percentage of effector T cells found in spleens and lymph nodes. In parallel, the percentage of CD4^+^CD25^+^FoxP3^+^ Treg population in the three compartments was reduced by the combinatorial strategies. Such reduction was much more significant in the PBMCs with a strong inverse correlation with animal survival. Mice which remained tumor negative (TN mice) showed the lowest percentage of Tregs along with the highest T_CD4_^+^/T_reg_ and T_CD8_^+^/T_reg_ ratio (Figure 2). Such results confirm previous observations showing that higher T_eff_/T_reg_ ratio directly correlate with better prognosis [33–35]. Unexpectedly, the most relevant effects were observed in animals receiving the vaccine combined with CI, alone or in combination with MCT. Indeed, while it has been repeatedly reported that metronomic chemotherapy reduces the number of regulatory T cells [8, 36, 37], this has not been previously shown for checkpoint inhibitors. A direct cytotoxic effect exerted by CI appears to be extremely unlikely. More plausible would be the down modulation of the FoxP3 expression in Tregs upon the binding of CI to the PD-1 molecule. The final result would be the observed dramatic reduction of FoxP3^+^ cells at flow cytometer analysis along with loss of their suppressive regulatory functions. Such hypothesis is currently under evaluation.

IFN-γ producing cells were found to be high in splenocytes of animals treated with vaccine combined to MCT, mainly focused on the wt Trp2 peptide. However, the strongest antigen-specific T cell response was observed in mice treated with the PEPT+MCT+CI combination, mainly focused on the two mutated peptides (Figure 3). The latter observation is in agreement with previous finding showing the strong correlation between efficacy of CI treatment and T cell response to tumor-associated mutated antigens [38]. A significant correlation was observed between animal survival and number of IFN-γ spots, which reached the highest values for the mutant peptides (Supplementary Figure 4). This correlated also with the inverse pattern of Tregs (Figure 4), confirming that the combination of such two immunological parameters is the key point in the observed inhibition of tumor growth as well as prolonged survival. As expected, tumors showed rare or absent immune cell infiltration confirming the poor immunogenicity of B16-derived lesions [30]. Moreover, the few infiltrating cells were confined at the peri-tumoral areas with no correlation with tumor progression, expressed as day of sacrifice (Figure 5). The role of such infiltrating cells in the remarkable outcome observed in the experiment remains unclear and needs to be further assessed.

TN mice showed a significant protection from a re-challenge with a second injection of B16F10 melanoma cells, without any further treatment. Notably, TN mice originally treated with the PEPT+MCT+CI combination showed prolonged delay in tumor growth in all animals, not statistically measurable due to the extremely limited number of animals. However, one of such animals (#4_PEPT+MCT+CI_) showed full protection remaining tumor-negative for the entire observation period (Figure 6B). Survival of the animals correlated with levels of Tregs (inverse correlation) as well as T_CD4_^+^/T_reg_ and T_CD8_^+^/T_reg_ ratio (direct correlation). The full-protected animal showed no increase in the percentage of Tregs and an astounding increase in the T_CD4_^+^/T_reg_ and T_CD8_^+^/T_reg_ ratio (>35.000) during the whole re-challenge experiment (Figure 6D, 6E). These results demonstrated that the effects of treatments administered in the first experimental step still persisted during the re-challenge experiment. Moreover, the protection correlated to the efficient immunological memory induced by the combinatorial treatments administered during the first experimental step and, in particular, by the PEPT+MCT+CI combination. Indeed, the number of IFN-γ producing splenocytes, already high at the day of re-challenge, were found considerably increased in the sacrificed mice showing a significant correlation with survival (Figure 7C). As for the first experimental step, the strongest reactivity against the mutated peptides was observed in mice previously treated with the combination including both MCT and CI. Within the latter group, the full protected mice showed the lowest number of IFN-γ producing splenocytes at the day of re-challenge but was characterized by the highest fold increase at the end of experiment focused on both mutated peptides and the pool of peptides (Figure 7B). The highest level of specific response to mutated peptides, together with the lowest percentage of Tregs, appeared to be the key immune correlates for the protection from the tumor re-challenge (Figure 8).

Overall, these results provide strong indications for the efficacy of combinatorial immunotherapy including a cancer vaccine, targeting mutated tumor-antigens, and immune modulatory treatments aiming at inhibiting immune suppressive cells (MCT) as well as blocking the immune checkpoints (CI). Although further confirmation on rechallenged animals is needed, the observed results overall suggest that such combinatorial immunotherapy protocol can be predicted to provide an improved clinical outcome in early-diagnosed cancer patients.

## MATERIALS AND METHODS

### Cell line and mice

C57BL/6 (H-2b MHC) female mice, 8 week old, were purchased from Harlan (Udine, Italy). All animals were housed at the Animal Facility of the Istituto Nazionale Tumori “Pascale” (Naples ,Italy). Mice were housed in number of 2-3 per cage and maintained in a conventional facility on a 12 hrs light:12 hrs dark cycle (lights on at 7:00 a.m.) in a temperature-controlled room (22 ± 2°C) and with food and water *ad libitum* at all times. The experimental protocols were in compliance with the European Communities Council directive (86/609/EEC) and were approved by the Italian Ministry of Health (approval number 835/2016).

Mouse melanoma B16F10 (ATCC, CRL-6323) cells were cultured in DMEM supplemented with 10% heat inactivated FBS, 100 U/ml penicillin and 100 mg/ml streptomycin (Invitrogen, Carlsbad, CA) at 37°C with 5% CO2. Cells were tested for mycoplasma before inoculation in mice (ATCC^®^, 30-1012K™).

### Subcutaneous tumor inoculation

Melanoma B16F10 cells were harvested in exponential growth phase by trypsinization and washed twice with ice-cold PBS, and then resuspended at a concentration of 1 × 10^6^ cells/ml. C57BL/6 mice were subcutaneously injected with 100 µl of B16F10 cells (1 × 10^5^ cells/mouse) on the right back flank. The tumor size was measured and documented every two days with a caliper, starting on day 7, and calculated using the formula (A × B^2^)/2 (A as the largest and B is the smallest diameter of tumor). Tumor growth was documented as mean tumor size with standard error. To record the survival of the tumor-bearing mice, either natural death or a tumor diameter greater than 1500 mm^3^ leading to death was counted as death.

### Multi-peptide vaccine and adjuvant

The multi-peptide cocktail vaccine used for the immunization including the melanoma-specific MHC class-I Tyrosinase-related protein 2 (Trp2_180–188_) and two MHC class-II mutated peptides with the mutated amino acid positioned centrally, MUT30 (Kinesin family member 18B, Kif18b - PSKPSFQEFVDWENVSPELNSTDQPFL) and MUT44 (cleavage and polyadenylation specific factor 3-like, Cpsf3l - EFKHIKAFDRTFANNPGPMVVFATPGM) previously described [29].

All peptide were synthesized at a purity of ≥ 80 % (Primm S.r.l., Milan Italy). The multi-peptide cocktail contained 100 µg per each peptide, emulsified with 50 μg of Polyinosinic:polycytidylic acid [poly(I:C); In*vivo*Gen] adjuvant formulated in PBS (200ul total volume), was subcutaneously (s.c.) injected.

### Immune modulating drugs administration

Cyclophosphamide (CTX) (Endoxan^®^, Baxter) (10 mg/Kg), Paclitaxel (PTX) (Taxol^®^, BMS) (5mg/Kg) and Docetaxel (DTX) (Taxotere^®^, Sanofi Aventis) (1mg/Kg) diluted with phosphate-buffered saline (PBS) were administered via intraperitoneal injection (i.p.). The dose was extrapolated to human equivalent dose (HED) according to Reagan-Shaw et al. [39]. Chemotherapy was weekly administered until the end of the experiment. An anti-mouse PD-1 MAb clone RMP1-14 (BioXCell, West Lebanon, NH USA) was used as checkpoint inhibitor (CI) and administered via intraperitoneal injection (i.p.) at a dose of 100 μg.

### Immunotherapy combinatorial protocols

Starting at seven days after implantation of B16F10 melanoma cells, C57BL/6 mice were weekly vaccinated by subcutaneous administration (s.c.) with peptide cocktail described before, alone (PEPT) or in combination with weekly metronomic chemotherapy (PEPT+MCT) as well as with CI (PEPT+CI). One group was treated with the peptide cocktail in combination with both MCT and CI (PEPT+wMCT+CI) (Supplementary Figure 1). Control mice were treated with endotoxin-free phosphate-buffered saline (PBS). Each experimental group consisted of six animals. At day 43, when only tumor-negative mice (TN mice) were still alive, the treatment was discontinued. The observation period was extended to day 77 (week 11), when the TN mice (8 mice) were re-challenged s.c. with same number of B16F10 melanoma cells injected in the left flank (opposite to the first one). Re-challenged mice were kept off from any immunological treatment.

At the time of sacrifice, whole blood was collected by puncture of the sinus retro-orbital vein prior analgesia with oxybuprocaine chlorhydrate. After euthanasia of mice spleens and tumor draining lymph nodes (LN) were resected and processed into single cell suspensions using a gentleMACS Dissociator (Miltenyi Biotec) according to the manufacturer’s instructions for immunological evaluation. Data were confirmed in two independent experiments.

### Antibodies for flow cytometry

PE-conjugated anti-mouse CD4 (clone RM4-5), PE/Cy7-conjugated anti-mouse CD8 (clone 53-6.7), FITC-conjugated anti-mouse CD25 (clone 3C7), Alexa Fluor 488-conjugated anti-mouse FoxP3 (clone 150D), antibodies were purchased from BioLegend (San Diego, CA). PerCP-eFluor 710-conjugated anti-mouse CD3 (clone 17-A2) was purchased from eBioscience (San Diego, CA).

### Characterization of effector and regulatory/suppressor cells in whole blood, in spleens and in tumor draining lymph nodes

CD4^+^, CD8^+^ T cells, CD4^+^CD25^+^FoxP3^+^ Tregs were characterized by flow cytometry in whole blood, spleens and tumor draining lymph nodes of each experimental group.

In particular, for Tregs detection, whole blood samples of each mouse were directly staining with PerCP-eFluor 710-conjugated anti-mouse CD3, PE-conjugated anti-mouse CD4 and FITC-conjugated anti-mouse CD25 and incubated for 30 min at 4°C. Following staining, whole blood was incubated with ACK lysing buffer for 7 min, washed with wash Medium (1 × PBS, 5% FBS, 0.1% NaN3), and after permeabilization, incubated with Alexa Fluor 488-conjugated anti-mouse FoxP3 for 30 min at 4°C in the dark. For T effector cells the blood samples were collected and directly staining with PerCP-eFluor 710-conjugated anti-mouse CD3, PE-conjugated anti-mouse CD4 (clone RM4-5) and PE/Cy7-conjugated anti-mouse CD8 (clone 53-6.7). After dissociation, splenocytes and LN were incubated with ACK lysing buffer for 7 min, washed and resuspended in RPMI medium and incubated for 30 min at 4°C in the dark with PE-conjugated anti-mouse CD4 and FITC-conjugated anti-mouse CD25. After washing and permeabilization, cells were incubated with Alexa Fluor 488-conjugated anti-mouse FoxP3 for 30 min at 4°C in the dark.

The staining was characterized by flow cytometry using Attune NxT hardware using Attune NxT v2.5 software (ThermoFisher, Waltham, MA USA).

### IFN-γ ELISpot assay

ELISPOT was performed according to BD Biosciences manufacturer instructions (BD ELISPOT Mouse IFN-γ ELISPOT Set cod. 551083). 5 × 10^5^ splenocytes were counted and plated in each well. Alternatively, 200 μl of whole blood were incubated with ACK lysing buffer for 7 min, washed and resuspended in RPMI medium. Cells were stimulated with 10 ug/ml of single and pool peptide used for the immunization and incubated for 24-26h. As negative and positive control, peptide diluents PBS and 5ug/ml of phorbol myristate acetate (PMA, Sigma-Aldrich) were used respectively. The plates were read with an AID EliSpot Reader Systems (AID GmbH, Strassberg, Germany). The results were calculated as spot forming counts as a mean of a duplicate count from the specific antigen stimulation minus the negative control.

### Analysis of tumor infiltrating lymphocytes (TIL) by immunohistochemistry (IHC)

Explanted tumors were analyzed by IHC. Tumors were formalin-fixed paraffin embedded (FFPE). Sections were stained using standard immunohistochemistry (IHC) techniques. Areas of necrosis or hemorrhage, identified on corresponding H&E-stained sections, were excluded from TIL scoring and two blinded individuals scored each section. 50-µm consecutive sections (3 per mouse) were stained for CD3 (clone SP7, Abcam), GZMB (clone 1, catalogue no. 50134-M08H, Sino Biologinal) and FoxP3 (polyclonal, catalogue no.NB100-39002, Novus Biologicals) and detected by a secondary HRP-conjugated antibody (Poly-HRP-anti-rabbit IgG, ImmunoLogic). Reaction was visualized by incubation with the peroxidase substrate (Vector Nova Red, Vector Laboratories). Each sample was analyzed by counting cells in 10 independent areas.

### Statistical analysis

Comparison between individual data points were performed with the unpaired two-sided Student’s *t*-test and ANOVA, as appropriate. Normally distributed data were represented as mean ± S.E.M. Two-way ANOVA and Bonferroni *post-hoc* analysis were used to examine the significance of differences among groups. All *P* values were two-tailed and considered significant if less than 0.05.

## SUPPLEMENTARY MATERIALS FIGURES



## Abbreviations

CI: anti-PD-1 checkpoint inhibitor
Cpsf3l: cleavage and polyadenylation specific factor 3-like
CTLA-4: Cytotoxic T-Lymphocyte Antigen 4
CTX: cyclophosphamide
DCs: dendritic cells
dMCT: daily metronomic chemotherapy
DTX: docetaxel
FBS: Fetal bovine serum
FFPE: formalin-fixed paraffin embedded
HED: human equivalent dose
IFN-γ: interferon gamma
IHC: immunohistochemistry
Kif18b: Kinesin family member 18B
LN: Limph node
MCT: metronomic chemotherapy
PBS: phosphate-buffered saline
PD -1: programmed death-1
PD-L1: programmed death ligand-1
PEPT: multi-peptide vaccine
PMA: phorbol myristate acetate
poly(I:C): Polyinosinic:polycytidylic acid
PTX: paclitaxel
TAA: tumor-associated antigens
Th1: T helper 1
TIL: tumor infiltrating lymphocytes
TME: Tumor microenvironment
Tregs: regulatory T cells
Trp2: Tyrosinase-related protein 2
