# Supplementary material for: Inhibition of tumor growth by cancer vaccine combined with metronomic chemotherapy and anti-PD-1 in a pre-clinical setting
Source: Oncotarget. 2017 Dec 8;9(3):3576–89. doi: 10.18632/oncotarget.23181 (PMC5790484; doi:10.18632/oncotarget.23181)
Supplement: Supplementary file 1 [file oncotarget-09-3576-s001.pdf]

Inhibition of tumor growth by cancer vaccine combined with metronomic chemotherapy and anti-PD-1 in a pre-clinical setting

SUPPLEMENTARY MATERIALS

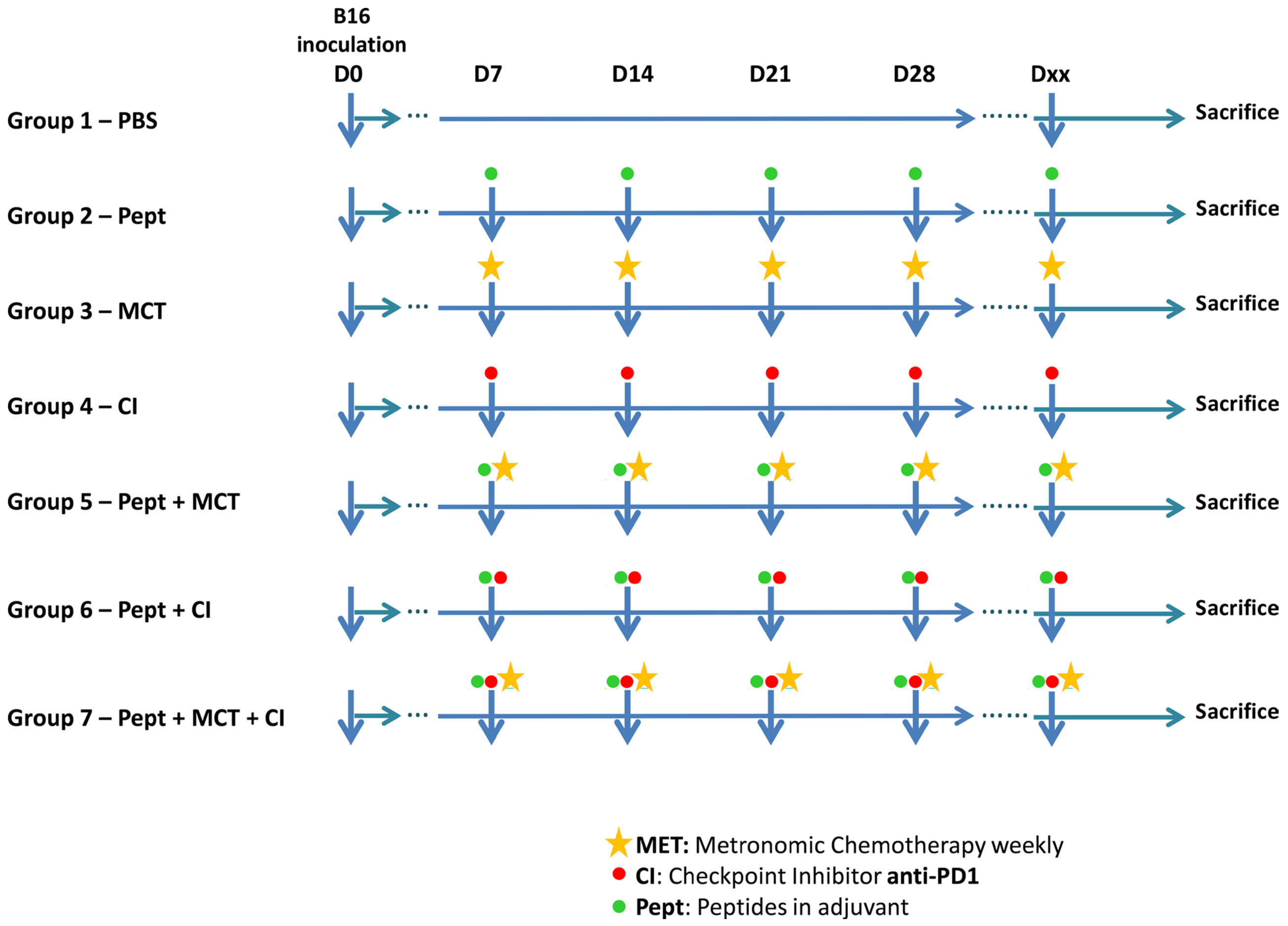

Supplementary Figure 1: Immunization protocol of the first experimental step.

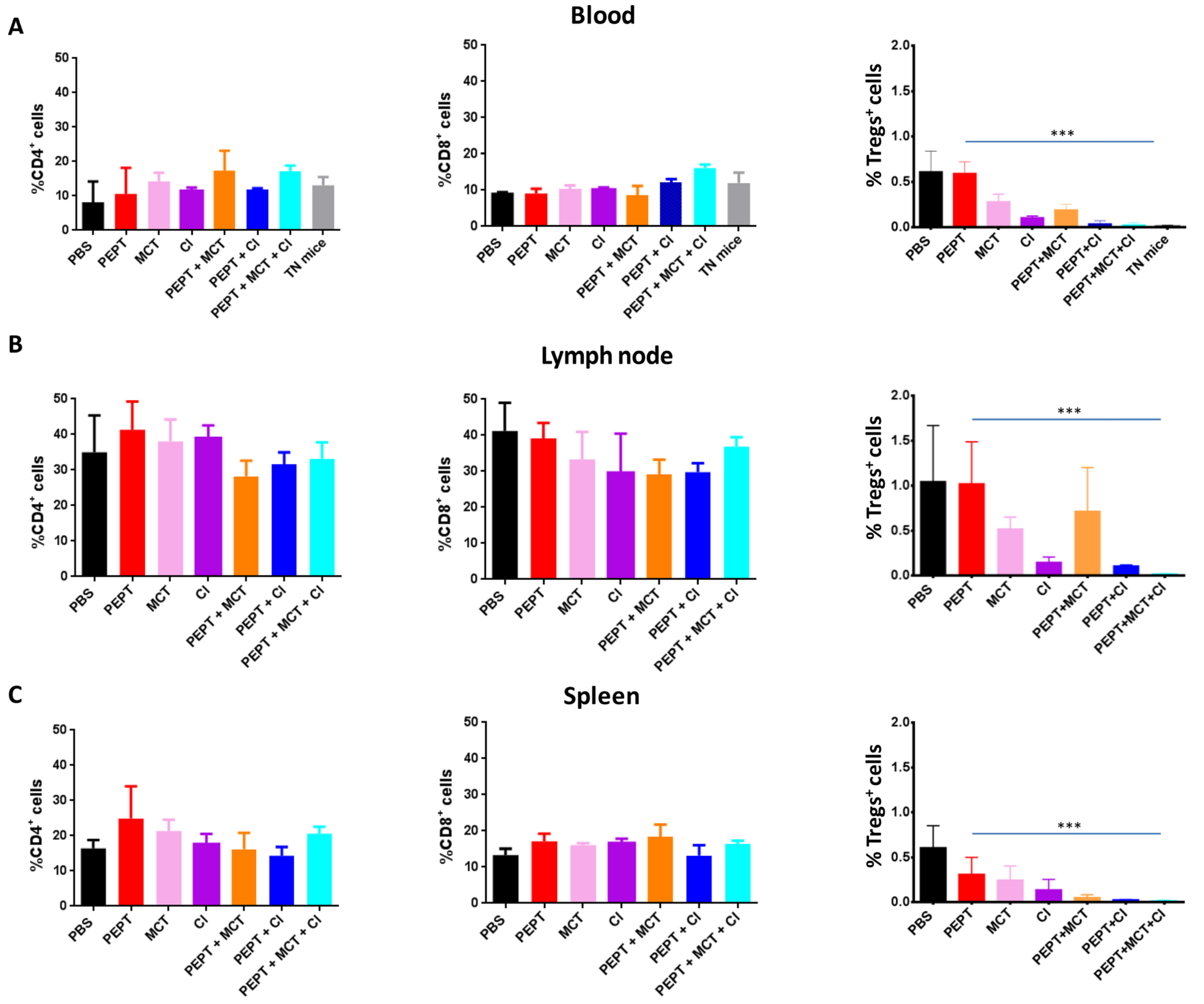

Supplementary Figure 2: Percentage of CD4+, CD8+ and CD4+ CD25+FoxP3+ T cells in the blood, lymph node and spleen districts represented as mean with SD in each experimental group.

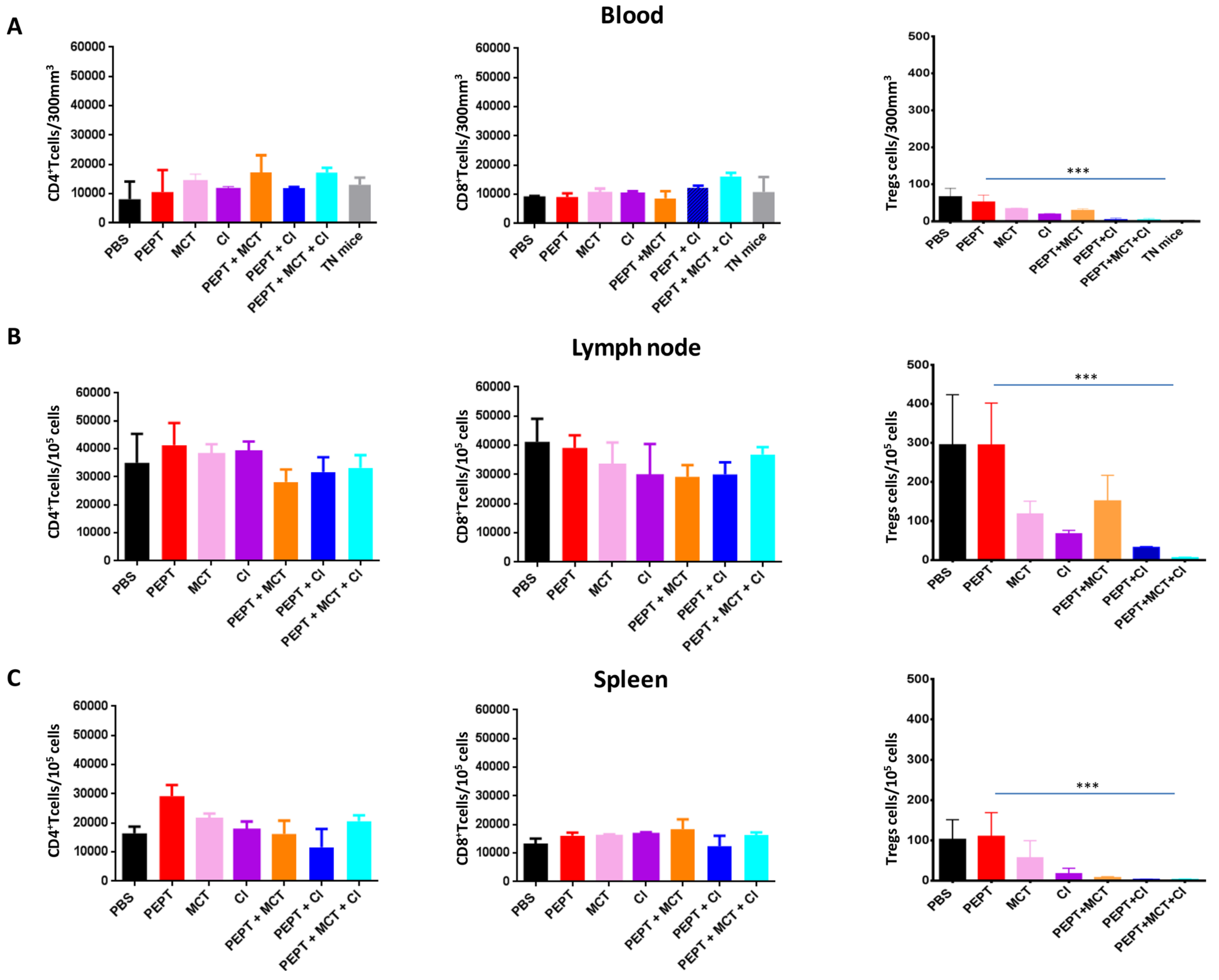

Supplementary Figure 3: Absolute numbers of CD4+, CD8+ and CD4+ CD25+FoxP3+ T cells in the blood, lymph node and spleen districts represented as mean with SD in each experimental group.

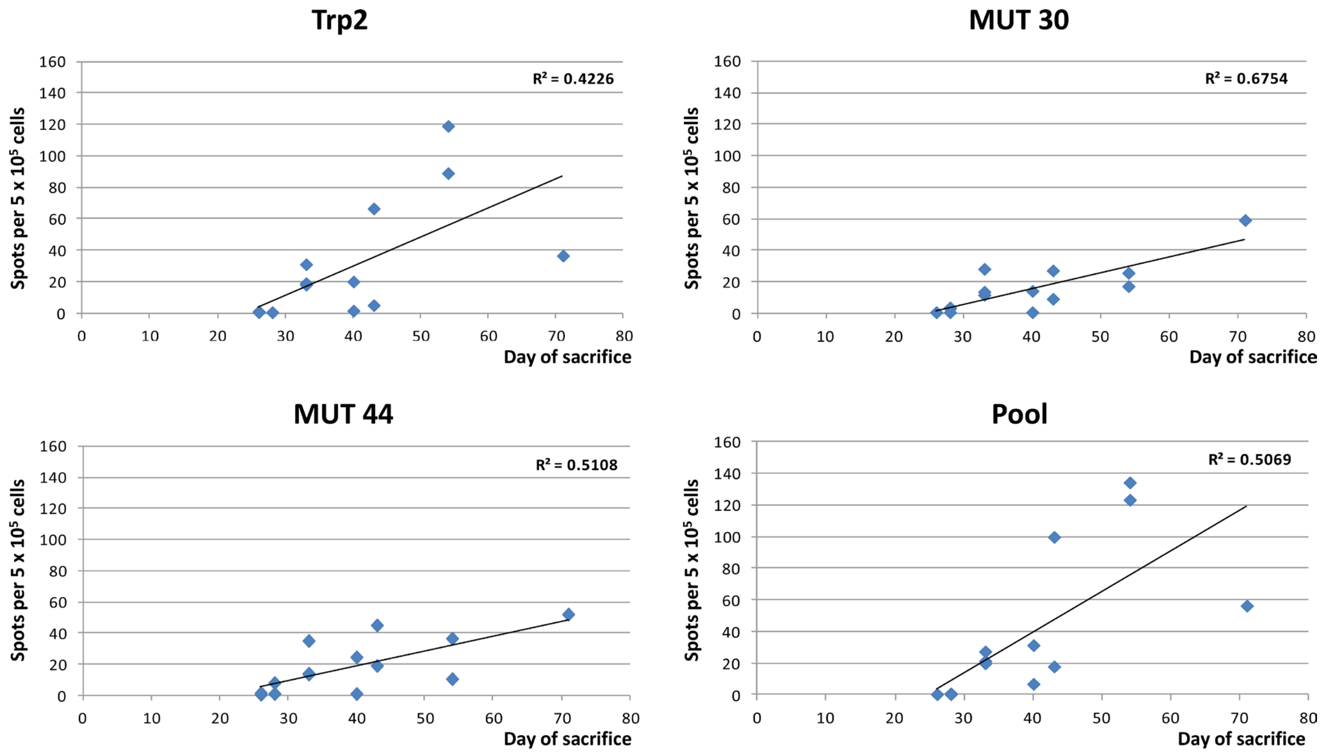

Supplementary Figure 4: Correlation analysis between number of spots per 5 × 10^5 splenocytes and day of sacrifice for each animal.
